# Novel insights into the molecular mechanism of amphisome-lysosome fusion

**DOI:** 10.1080/27694127.2022.2132446

**Published:** 2022-10-11

**Authors:** Yukako Oe, Tamotsu Yoshimori, Shuhei Nakamura

**Affiliations:** a Department of Intracellular Membrane Dynamics, Graduate School of Frontier Biosciences, Osaka University, Osaka, Japan; b Department of Genetics, Graduate School of Medicine, Osaka University, Osaka, Japan; c Institute for Advanced Co-Creation Studies, Osaka University, Osaka, Japan

**Keywords:** Aggrephagy, amphisome, autophagy, lysophagy, lysosome, PACSIN1

Macroautophagy, hereafter referred to as autophagy, is an evolutionarily conserved intracellular bulk degradation system, which maintains cellular homeostasis by promoting the clearance of long-lived proteins, damaged organelles, and aggregated proteins. These functions are critical to prevent various human diseases including neurodegenerative diseases. During autophagy, double-membrane phagophores surround cytoplasmic materials, maturing into autophagosomes and either fusing with endosomes, which generates transient structures called amphisomes, and then lysosomes, or autophagosomes directly fuse with lysosomes. Finally, in both processes, autolysosomes are generated and degrade their contents through the action of lysosomal hydrolases. Although the presence of these two fusion paths is recognized mainly based on electron microscopy studies, the molecular mechanism and biological significance of these two paths remain largely unclear.

In our recent study [1], we found that PACSIN1 as a novel autophagy regulator, which mediates the amphisome-lysosome fusion process. PACSINs are a family of cytoplasmic phosphoproteins that play a crucial role in intracellular vesicle trafficking, cytoskeletal rearrangement, caveolar biogenesis, neuronal development, and cell migration. However, it remains unknown whether PACSIN1 acts in autophagy. First, we revealed that *PACSIN1* KO cells show accumulation of autophagosomes due to a decrease of autophagic activity. Intriguingly, *PACSIN1* KO cells show a decrease of autophagic activity only under nutrient-rich conditions, not under starved conditions, suggesting that PACSIN1 is required for basal autophagy but not starvation-induced autophagy. Because *PACSIN1* KO cells show normal lysosome activity, accumulation of autophagosomes in *PACSIN1* KO cells can be derived from a defect in autophagosome-lysosome fusion. Indeed, we confirmed that the fusion process is impaired through a tandem fluorescent-tagged LC3 (mRFP-EGFP-LC3) assay, which allows us to monitor autophagic flux based on the differential pH stability of EGFP and mRFP. To our surprise, electron microscopy analysis revealed that amphisomes rather than autophagosomes accumulate in *PACSIN1* KO cells. Consistent with the characteristics of amphisomes, which are the hybrid structure of autophagosomes and late endosomes, the autophagosome marker, LC3 is well colocalized with the late endosome marker CD63 in *PACSIN1* KO cells compared to WT cells. These results suggest that PACSIN1 regulates the amphisome-lysosome fusion process.

Previously, it has been shown that autophagosome-endo/lysosome fusion is mediated by two autophagic SNARE complexes, which are STX17-SNAP29-VAMP8 and YKT6-SNAP29-STX7, and some tethering factors. Thus, we wondered if PACSIN1 regulates the amphisome-lysosome fusion process via any of these fusion factors. We found that PACSIN1 is required for the assembly of both autophagic SNARE complexes. In addition, PACSIN1 interacts with SNAP29, which is a common component of these SNARE complexes. These results indicate that PACSIN1 might promote the assembly of the STX17 and YKT6 SNARE complexes through interaction with SNAP29. Further analyses are necessary to understand how PACSIN1 regulates specific fusion between amphisomes and lysosomes via the assembly of these common autophagic SNAREs.

Intriguingly, we revealed that PACSIN1 is required for basal autophagy but not starvation-induced autophagy. This suggests the possibility that two autophagic routes, which are the amphisome fusion path and the direct fusion path, are utilized depending on specific stress and/or physiological conditions. To test this possibility, we further analyzed whether the PACSIN1-dependent fusion path is required for selective autophagy, which targets different substrates including damaged mitochondria (mitophagy), lysosomes (lysophagy) and aggregated proteins (aggrephagy) induced by different stimuli. We found that PACSIN1 is required for lysophagy and aggrephagy, but not mitophagy. This suggests that the PACSIN1-depedent fusion path is preferentially utilized for subsets of selective autophagy. Finally, we confirmed a conserved role of PACSIN1 in autophagy in *C. elegans*. Deletion of *sdpn-1*, a homolog of *PACSINs*, shows impairment of basal autophagy and clearance of aggregated protein in *C. elegans*. Taken together, the amphisome-lysosome fusion path is preferentially utilized depending on specific stress and/or physiological conditions, and PACSIN1 acts as a conserved key regulator for this specific fusion process.

**Figure 1.**
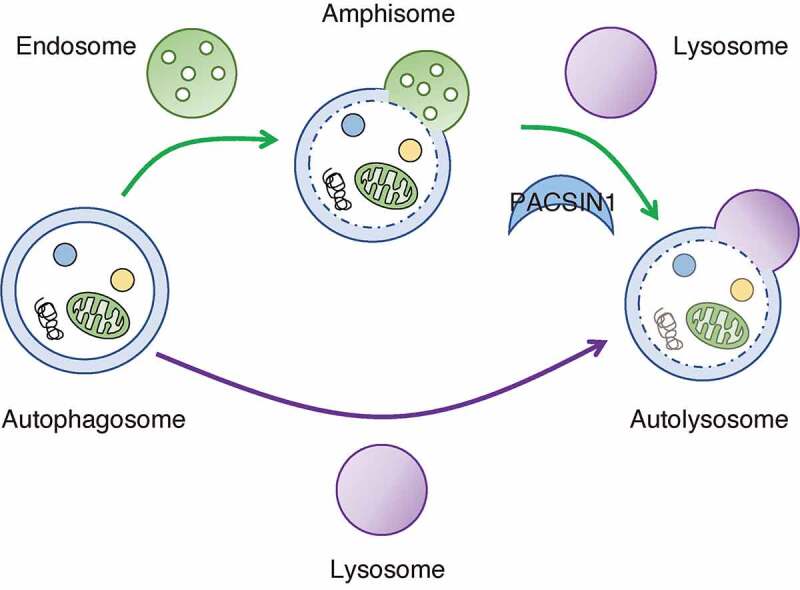
Model of two autophagosome-lysosome fusion paths. PACSIN1 regulates amphisome-lysosome fusion via promoting autophagic SNARE assembly. The PACSIN1-dependent amphisome-lysosome fusion path is preferentially selected over the direct fusion path in basal autophagy and subsets of selective autophagy, such as lysophagy and aggrephagy.

Collectively, we discovered that PACSIN1 regulates the amphisome-lysosome fusion process through promoting autophagic SNARE assembly. In addition, our studies on the roles of PACSIN1 reveal that the two autophagic fusion pathways are preferentially selected depending on the cellular context (Figure 1). We showed that the PACSIN1-dependent amphisome-lysososme fusion path is preferentially selected over the direct fusion path in basal autophagy. Conversely, in starvation-induced autophagy the direct fusion path might be preferentially selected. Importantly, while one route is preferentially selected depending on the context, another route is not completely shut off. In this context, determination of the preferential route might depend on autophagic activity or lysosomal activity because basal autophagy is characterized by relatively low autophagic activity and lysosomal activity compared to the starved condition. In addition, a nutrient-signal might regulate the fusion path. Furthermore, some types of selective autophagy, in particular lysophagy and aggrephagy, but not mitophagy, depend on the amphisome-mediated fusion path, implying that determination of the preferential route might depend on the degraded cargo specificity. To identify what kinds of signals determine the selection of the autophagy fusion path is of particular interest. Additionally, the difference in the role of the two paths also needs to be clarified in a future study. Due to low degradative capacity, amphisomes may act as a compartment to temporarily sequester the substrate from degradation. Another possibility is that the amphisome-mediated fusion path can select the route not only for degradation but also secretion. Therefore, the preferential availability of different fusion paths may mediate temporal and spatial regulation of substrate degradation in response to the context.
